# Electroencephalography theta/beta ratio covaries with mind wandering and functional connectivity in the executive control network

**DOI:** 10.1111/nyas.14180

**Published:** 2019-07-16

**Authors:** Dana van Son, Mischa de Rover, Frances M. De Blasio, Willem van der Does, Robert J. Barry, Peter Putman

**Affiliations:** ^1^ Institute of Psychology Leiden University Leiden the Netherlands; ^2^ Leiden Institute for Brain and Cognition Leiden the Netherlands; ^3^ Brain and Behaviour Research Institute and School of Psychology University of Wollongong Wollongong Australia

**Keywords:** mind wandering, EEG, executive control, default mode network, controlled thought

## Abstract

The ratio between frontal resting‐state electroencephalography (EEG) theta and beta frequency power (theta/beta ratio, TBR) is negatively related to cognitive control. It is unknown which psychological processes during resting state account for this. Increased theta and reduced beta power are observed during mind wandering (MW), and MW is related to decreased connectivity in the executive control network (ECN) and increased connectivity in the default mode network (DMN). The goal of this study was to test if MW‐related fluctuations in TBR covary with such functional variation in ECN and DMN connectivity and if this functional variation is related to resting‐state TBR. Data were analyzed for 26 participants who performed a 40‐min breath‐counting task and reported the occurrence of MW episodes while EEG was measured and again during magnetic resonance imaging. Frontal TBR was higher during MW than controlled thought and this was marginally related to resting‐state TBR. DMN connectivity was higher and ECN connectivity was lower during MW. Greater ECN connectivity during focus than MW was correlated to lower TBR during focus than MW. These results provide the first evidence of the neural correlates of TBR and its functional dynamics and further establish TBR's usefulness for the study of executive control, in normal and potentially abnormal psychology.

## Graphical abstract

The aim of our current study was to further clarify the relations between resting‐state theta/beta ratio (TBR) and TBR's dynamic relation with states of increased/decreased cognitive control and their neurobiological underpinning in terms of executive control network/default mode network connectivity. We assessed mind wandering and focused attention during EEG and fMRI measurements in the same participants on 2 separate days, exploiting TBR's excellent retest reliability.

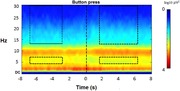

## Introduction

Resting‐state electroencephalography (EEG) provides measures of neural oscillatory activity in different frequency bands, such as the slow theta (4–7 Hz) and faster beta (13–30 Hz). Lubar^1^ reported higher theta/beta ratio (TBR) in attention‐deficit hyperactivity disorder (ADHD) and attention deficit disorder, which has been frequently replicated since (e.g., see Refs. 2, 3, 4). Research into the relation between TBR and AD(H)D has remained largely descriptive, however—with the exception of studies that demonstrated that the administration of catecholamine agonists is therapeutic in AD(H)D through the restoration of suboptimal prefrontal cortical control (i.e., normalizing TBR^2^, ^5^, ^6^, ^7^, ^8^). This further suggests that high TBR scores may reflect the (frontal) cortical hypoactivity, which characterizes these disorders (e.g., Ref. 9).

The functional cognitive significance of TBR has been further investigated in non‐AD(H)D samples. This research indicated that although high TBR seems to indicate lower attentional capacity, TBR more likely reflects a continuum of executive cognitive processing efficiency, rather than being a marker of a particular disorder. For instance, TBR is negatively related to functions requiring prefrontal executive control (EC): modulation of response inhibition in an emotional go/no‐go task^10^ and downregulation of negative affect.^11^ In healthy samples, TBR correlated negatively with self‐reported trait^10^, ^12^, ^13^, ^14^ and state attentional control (AC)^12^ and with the controlled modulation of threat‐selective attention.^14^, ^15^, ^16^ TBR also correlated negatively with objectively measured AC.^17^ Higher TBR has also been related to more reward‐motivated decision making that requires executive reversal learning and inhibition of dominant approach−motivated behavior^18^, ^19^, ^20^ and to lower trait anxiety,^10^, ^14^, ^15^ also implying potential benefits of having a higher TBR. Taken together, these studies demonstrate that TBR is related to a variety of psychological functions that require prefrontal executive regulation of emotional and motivational processes that are likely subcortically mediated. Almost all previous studies examining TBR in relation to executive processes assessing healthy participants focused on frontal TBR, which is also the focus of the current study.^10^, ^11^, ^12^, ^13^, ^14^, ^15^, ^16^, ^18^, ^21^, ^22^

TBR is typically measured during several minutes of resting state, without manipulation of executive processes. Consequently, the evidence that TBR reflects EC functions remains indirect. It is unclear exactly which processes these relations reflect or what are TBR's neurological underpinnings. A more thorough understanding would require continuous measurement of TBR during the execution of experimentally identified psychological functions.

The processes related to TBR, including threat‐selective attention, are not restricted to attentional processing of external stimuli. “Mind wandering” (MW^23^) occurs when thoughts are not controlled by top‐down processes, such as AC.^24^, ^25^ MW is a predictor of processes, like prospection and future planning,^26^, ^27^ creativity,^28^ and “mental breaks” remediating an unpleasant mood,^29^ but also of performance errors^30^ and poor executive cognitive control.^25^, ^31^, ^32^ Consequently, the frequently observed relation between resting‐state TBR and indices of executive cognitive control might reflect more frequent or prolonged episodes of MW occurring during the resting‐state measurement in people with low AC. The current study focuses on MW as related to reduced AC for task performance.

Higher EEG theta and lower EEG beta band power have been observed during states of MW compared with focused attention.^33^ Participants in that study were asked to press a button when they realized that their mind had wandered off a breath‐counting task. Higher TBR occurred during a 6‐s window just before the button press, and lower TBR during a 6‐s window just after the button press when participants refocused on their breath counting. We recently replicated this study and similarly found higher frontal TBR during the MW episodes compared with the task‐focused periods.^34^ These results support a hypothesis that relations between resting‐state TBR and EC might reflect the brain dynamics, which occur when participants engage in MW or related states of reduced cognitive control during the resting‐state measurement. This warrants a comparison between EEG‐based TBR and functional magnetic resonance imaging (fMRI)−based localization of the corresponding cortical and subcortical activity.

fMRI studies have revealed that areas, including the posterior cingulate cortex (PCC), medial prefrontal cortex (mPFC), parahippocampal gyrus (PHG), and angular gyrus (AG), are active during MW.^35^, ^36^ These areas are jointly referred to as the default mode network (DMN^37^). Functional connectivity within this network is high during task‐irrelevant thoughts,^32^ and is related to MW^38^, ^39^, ^40^ and also to ruminative thoughts.^41^ Moreover, it has been reported that the dorsolateral PFC, dorsal anterior cingulate cortex (ACC), and posterior parietal regions became active during awareness of MW, during subsequent attentional shifting back to task performance, and during subsequent sustained attention in a breath‐counting task.^35^, ^40^ These brain regions are elements of the so‐called executive control network (ECN^42^). The ECN is active during cognitive tasks involving demanding top‐down processes, including working memory (WM), mental calculation, and spatial WM,^43^ and this network is associated with goal‐directed AC.^42^, ^44^, ^45^

In summary, states of MW versus controlled attention have been associated with increased TBR^33^, ^34^ and with decreased activity in brain areas that are involved in EC,^35^ but in separate studies. Together, these findings support the hypothesis that low TBR reflects a state of increased top‐down cognitive control, involving functional connectivity in the ECN, whereas high TBR reflects uncontrolled thought and functional connectivity in the DMN. The aim of the current study was to further clarify the relations between resting‐state TBR and TBR's dynamic relation with states of increased/decreased cognitive control and their neurobiological underpinning in terms of ECN/DMN connectivity. We assessed MW and focused attention during EEG and fMRI measurements in the same participants on 2 separate days, exploiting TBR's excellent retest reliability.^13^, ^17^

We tested the following hypotheses: (1) Frontal TBR is higher during MW episodes than during focused episodes, and this MW‐related change is related to resting‐state (i.e., baseline) frontal TBR. We also explored changes in the EEG delta and alpha bands, as MW‐related changes in these bands were observed in van Son *et al*.^34^ and Braboszcz and Delorme.^33^ (2) MW‐related changes in frontal TBR mediate a relationship between resting‐state frontal TBR and AC. (3) Functional connectivity within the ECN is stronger during focused episodes than during MW episodes, with the opposite pattern of functional connectivity within the DMN. (4) MW‐related EEG changes are positively correlated with MW‐related changes of the functional connectivity within the DMN and negatively with changes of connectivity in the ECN.

## Methods

### Participants

Eighty‐four right‐handed participants between 18 and 32 years (35 men) were recruited from Leiden University. Exclusion criteria were factors that would likely adversely affect EEG, MRI, or attention, including severe physical or psychological dysfunction, and/or the use of psychotropic medication, and having typical contraindications for MRI scanning. Baseline resting‐state TBR and MW‐related EEG were assessed during the first session, and only those participants who reported 25 or more MW episodes were invited to return for a second session on a separate day to perform the same task in the MRI scanner. This selection criterion was used to increase the chance of obtaining enough button presses during MRI testing for reliable fMRI analysis (defined a priori as ≥15 MW episodes). Informed consent was obtained prior to testing, and participants received a monetary reimbursement of €15 at the end of each session to compensate them for their participation. The study was approved by the Medical Ethics Committee of Leiden University Medical Center (LUMC).

## Materials

### Questionnaires

Participants completed the trait version of the State‐Trait Anxiety Inventory (STAI‐T^46^) and the attentional control scale (ACS^47^). The STAI‐T assesses trait anxiety (20 items, range 20–80; Cronbach's alpha in the current study = 0.89) with items like “I feel nervous and restless” and “I have disturbing thoughts” on a four‐point Likert scale. The ACS assesses self‐reported AC in terms of attentional focus, attentional switching, and the capacity to quickly generate new thoughts (20 items, range 20–80; Cronbach's alpha in the present study = 0.86), with items like “I can quickly switch from one task to another” and “I have a hard time concentrating when I'm excited about something.”

### Breath‐counting task

The breath‐counting task was as in van Son *et al*.;^34^ based on Braboszcz and Delorme.^33^ Participants were asked to count their breath cycles (one inhalation and one exhalation) from 1 to 10 and then start from 1 again (with eyes closed). They were instructed to press a button whenever they realized they had stopped counting, continued counting further than 10, or had to reflect intensively on what the next count was. Prior to performance of the task, participants were instructed to bring their focus back to breath‐counting after pressing the button. To retain consistency with the procedure of Braboszcz and Delorme,^33^ and subsequently van Son *et al*.,^34^ a passive auditory oddball was presented during the task and debriefing questions were presented at the end of each block as it is possible that this might influence the occurrence of MW episodes. The oddball‐related EEG and fMRI data were not of interest here and so participants were instructed to ignore the tones. The responses to the debriefing questions were analyzed only for the reported nature of off‐task thoughts.

### EEG recording

Continuous EEG was measured from 31 Ag/AgCl electrodes located according to the 10–20 system, using an ActiveTwo BioSemi system (BioSemi, the Netherlands). Electrodes were also placed on the left and right mastoids for offline rereferencing. EEG data were collected at a sampling rate of 1024 Hz and amplified with a gain of 16× at a bandwidth between DC‐400 Hz, and were downsampled to 256 Hz for offline processing.

### MRI recording parameters

A whole‐brain 3D T1–weighted structural scan, two task scans (T2^*^–weighted echo‐planar images; EPIs), and a B0 field map were acquired using a 3‐T Philips Achieva scanner equipped with a 32‐channel head coil. The T1‐weighted scan (field of view (FOV): 224 × 177.33 × 168 mm; 140 slices; in‐plane voxel resolution = 0.88 × 0.88 mm; slice thickness = 1.2 mm; TR: 9.8 ms; TE: 4.59 ms; flip angle 8°; acquisition matrix: 192 × 192; scan duration: 5 min) was used for registration to the standard 2‐mm MNI152 template image. The task scans consisted of 542 whole brain T2^*^‐weighted EPIs (FOV: 220 × 114.7 × 220 mm; 38 slices; in‐plane voxel resolution = 2.75 × 2.75 mm; slice thickness = 2.75 mm + 0.275 mm slice gap; TR: 2200 ms; TE: 30 ms; flip angle 80°; acquisition matrix: 80 × 80; scan duration: 20 min each). The B0 field map (parameters were as T2^*^‐weighted EPIs, except TR: 200 ms; TE: 3.21 ms; flip angle 30°; scan duration: 65.6 s) was used to undistort the task scans.

## Procedure

### General procedure

During the first session, informed consent was obtained and participants completed the ACS and STAI‐T. EEG equipment was then fitted and used to measure activity during a 10‐min resting‐state with eyes closed, and then during the breath‐counting task that comprised two 20‐min blocks (40 min in total) with a ∼2‐min break between. Participants who reported ≥25 MW episodes in this session were invited to participate in a second session within 7 days, wherein participants repeated the breath‐counting task during MRI acquisition.

## Data reduction

### Defining epochs for MW and focused attention

Previous studies^33^, ^34^ analyzed the −8‐ to −2‐s window prior to the button press as MW episodes, and the 2‐ to 8‐s window following the button press as focused attention. However, due to the reduced temporal precision of MRI data acquisition (a repetition time (TR) of 2.2 s), we selected only those TRs that fitted fully within those windows for fMRI hypothesis‐testing. This resulted in the selection of a pre‐button press MW window of −7.1 to −2.7 s and a post‐button press focused attention window of 1.7 to 6.1 s (thus two TRs each, corresponding to the TR windows of fMRI data of −1.1 to 3.3 s and 7.7 to 12.1 s when taking into account the standard 6 s for the hemodynamic response function (HRF)). These narrower epochs were therefore used to quantify the MW (i.e., −7.1 to −2.7 s) and focused attention (i.e., 1.7 to 6.1 s) windows for both the EEG and fMRI data, facilitating their joint analysis.

### EEG spectral composition: resting‐state

For all EEG analyses, frontal EEG measures were calculated by averaging the data from F3, Fz, and F4 positions. Resting‐state EEG data were rereferenced offline to the linked mastoids and automatically corrected for ocular artifacts^48^ in segments of 4 s using BrainVision Analyzer V2.04 (Brain Products GmbH, Germany). Baseline resting‐state EEG was then subjected to a fast Fourier transformation (Hamming window length 10%) to calculate power density in the theta (4–7 Hz) and beta (13–30 Hz) bands. TBR was calculated by dividing the power density in theta by that in beta. Baseline EEG values were nonnormally distributed and were therefore log‐normalized with a log10 transformation.

### EEG spectral composition: breath‐counting task

The EEG data recorded during the breath‐counting task were similarly preprocessed in Brain Vision Analyzer V2.0.4 (Brain Products GmbH). This was used to rereference the data offline, apply an ocular correction, interpolate bad channels, and extract single‐trial epochs for 8.25 s pre‐ to 8.25 s post‐each button press. The remaining data quantification was completed within MATLAB (Mathworks®, Version 8.0.0.783, R2012b) using EEGLAB (Version 13.4^49^) and custom scripts.

Event‐related spectral perturbation (ERSP) data were derived at each site for each participant using 257 applications of a 500‐ms (128 points) sliding discrete Fourier transform (DFT) window. The data in each window were DC corrected, multiplied by a 10% Hanning window, and zero‐padded to 1 s (256 points) prior to the application of the DFT and a subsequent correction was applied for the use of the Hanning window. This yielded absolute power ERSP data from –8 to +8 s relative to the button press at 1‐Hz spectral resolution and 62.5‐ms temporal resolution. Mean (across button press) ERSP data were computed within‐subjects, and the associated mean ERSP band powers were derived at each site for delta (1–3 Hz), theta (4–7 Hz), alpha (8–12 Hz), and beta (13–30 Hz) during the 4.4‐s MW (−7.1 to −2.7 s) and focused attention (1.7 to 6.11 s) windows of interest. These data were not normally distributed and were therefore normalized with a log10 transformation prior to analysis.

### fMRI analysis

Data were preprocessed using FSL version 5.0.7 (FMRIB's Software Library, http://www.fmrib.ox.ac.uk/fsl). First, brain extraction tool (BET as implemented in FSL) was used to subtract nonbrain tissue from the structural images. Next, all task data (the EPIs) were motion corrected, corrected for field inhomogeneities (B0‐unwarping), high‐pass filtered (100 s), registered first to the structural image (BBR), and then to the MNI152 template image (12 dof), and spatially smoothed using a 5‐mm full width at half maximum (FWHM) Gaussian kernel. Probabilistic independent component analysis^50^ was carried out using MELODIC (Multivariate Exploratory Linear Decomposition into Independent Components) Version 3.05 as implemented in FSL. The preprocessed task data of all participants were decomposed into 15 components. The components representing DMN and ECN networks were selected based on Smith *et al*.^51^ The set of spatial maps from the DMN component and ECN component was used to generate subject‐specific versions of the spatial maps, and associated time series, using dual regression.^50^ For each subject, the set of spatial maps was regressed per component (as spatial regressors in a multiple regression) into the subject's 4D space–time dataset, resulting in a set of subject‐specific time points, one set of beta values for each component. The beta values for the DMN and ECN components were selected for further analysis. All beta values were normalized by subtracting the average of each value per brain network and then log10 transformed to correct for skewness.

### Participants

Of the 84 participants who completed the first session, 56 participants reported 25 or more MW episodes and were invited to participate in the second session within 7 days (mean number of days between sessions = 2.8, SD = 1.9; range 1–7 days). Of those, data from a number of participants had to be discarded because of excessive (movement) artifacts around button presses in the EEG. Based on Braboszcz and Delorme^33^ and van Son *et al*.,^34^ and exploration of MW frequencies in the current data, we chose to require a minimum of 11 clean EEG epochs for analysis, resulting in complete EEG and fMRI data for 27 participants (16 males). These participants had a mean age of 24.7 (SD = 2.7 range: 18–30) years. Their mean ACS score was 51.70 (SD = 7.83, range 39–69), and their STAI‐T score was 39.15 (SD = 8.95, range 26–60). One participant had raw EEG theta and beta values more than 3 SD above the mean in the breath‐counting task; the participant's data were therefore omitted from this study.

## Results

### Hypothesis I: changes in frontal TBR and baseline TBR

**Figure 1:**
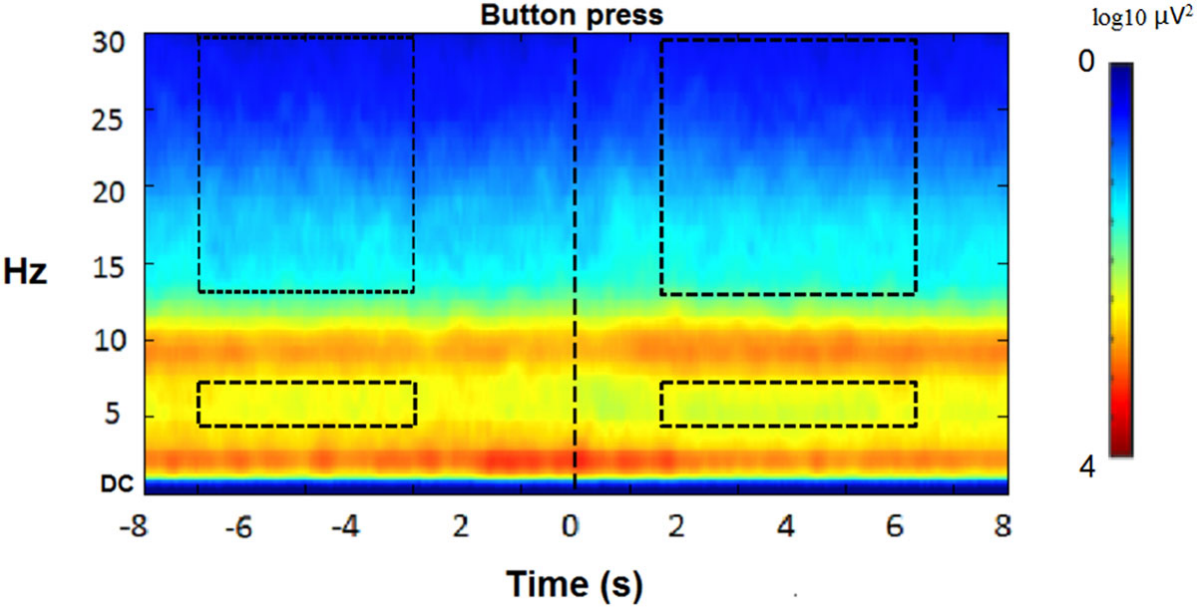
ERSP spectral plot of the frontal average (across F3, Fz, and F4 sites) at 1‐Hz frequency resolution and 62.5 ms time resolution. Rectangular frames highlight the epochs of primary interest corresponding to the two “real time” 2‐TR epochs that fall within the predefined periods for MW and focused attention (the upper high‐frequency frames are for beta and the lower are for theta).

In the breath‐counting task, the remaining 26 participants had between 21 and 92 assessed button presses during the EEG session (*M* = 47.96, SD = 20.54), and between 15 and 115 assessed button presses during the MRI session (*M* = 49.41, SD = 21.55). Number of assessed button presses did not differ significantly between these sessions (within‐subjects), *t*(25) = 0.36, *P* = 0.723, but were significantly correlated (*r*(24) = 0.51; *P* = 0.007). The grand mean frontal ERSP data (across F3, Fz, and F4) are visualized in Figure 1 for this task. Mean frontal ERSP data (across F3, Fz, and F4) in the pre‐ and post‐button press windows of interest, representing MW and focused episodes, respectively, were assessed using paired samples *t*‐tests; these analyses were conducted independently for the theta and beta bands, and for the TBR. As seen in Figure 1, theta power was significantly higher during the MW (pre) than focused (post) episodes (*t*(25) = 2.38, *P* = 0.025, *d* = 0.47), and beta was significantly lower during the MW (pre) compared with focused (post) episodes (*t*(25) = −3.79, *P* = 0.001, *d* = 0.74). TBR was confirmed to be significantly higher during MW (pre) compared with focused (post) episodes (*t*(25) = 5.72, *P* < 0.001, *d* = 1.13), and these values (TBR in MW and focused attention) were highly correlated (*r*(25) = 0.93, *P* < 0.001).

TBR of the mean frontal resting‐state power densities (across F3, Fz, and F4) in these same participants was 1.09 (SD = 0.60, range 0.35–3.06 (raw, unnormalized values)). Frontal resting‐state TBR was correlated marginally with the MW‐related change in frontal TBR (i.e., MW minus focused frontal TBR, or pre‐ minus post‐button press); *r*(24) = 0.35, *P* = 0.078. That is, higher resting or baseline TBR predicted a greater difference in TBR between MW relative to focus periods. Together, these findings confirm hypothesis I.

Additional post‐hoc paired‐samples *t*‐tests were conducted to test changes in frontal alpha and frontal delta band power for the same MW (pre) versus focused (post) episodes. Frontal alpha power was significantly higher during focused episodes compared with MW episodes (*t*(25) = −3.19, *P* = 0.004, *d* = 0.63), although delta showed no significant change between the MW and focused attention episodes (*t*(25) = 1.62, *P* = 0.117, *d* = 0.32).

### Hypothesis II: baseline frontal TBR and AC, mediated by changes in TBR

Pearson correlation was used to test for a relationship between frontal resting‐state TBR and ACS. This correlation was not significant (*r*(24) = −0.14, *P* = 0.51), and remained nonsignificant when controlling for STAI‐T (c.f. Refs. 10, 12, 13, 14); *r*(23) = −0.03, *P* = 0.90. Consequently, hypothesis II was not supported. Additional analyses revealed that resting‐state frontal TBR was correlated positively with STAI‐T score (*r*(24) = 0.43, *P* = 0.029), and this relationship remained significant when controlling for ACS (*r*(23) = 0.41, *P* = 0.041).

### Hypothesis III: changes in DMN and ECN functional connectivity

**Figure 2:**
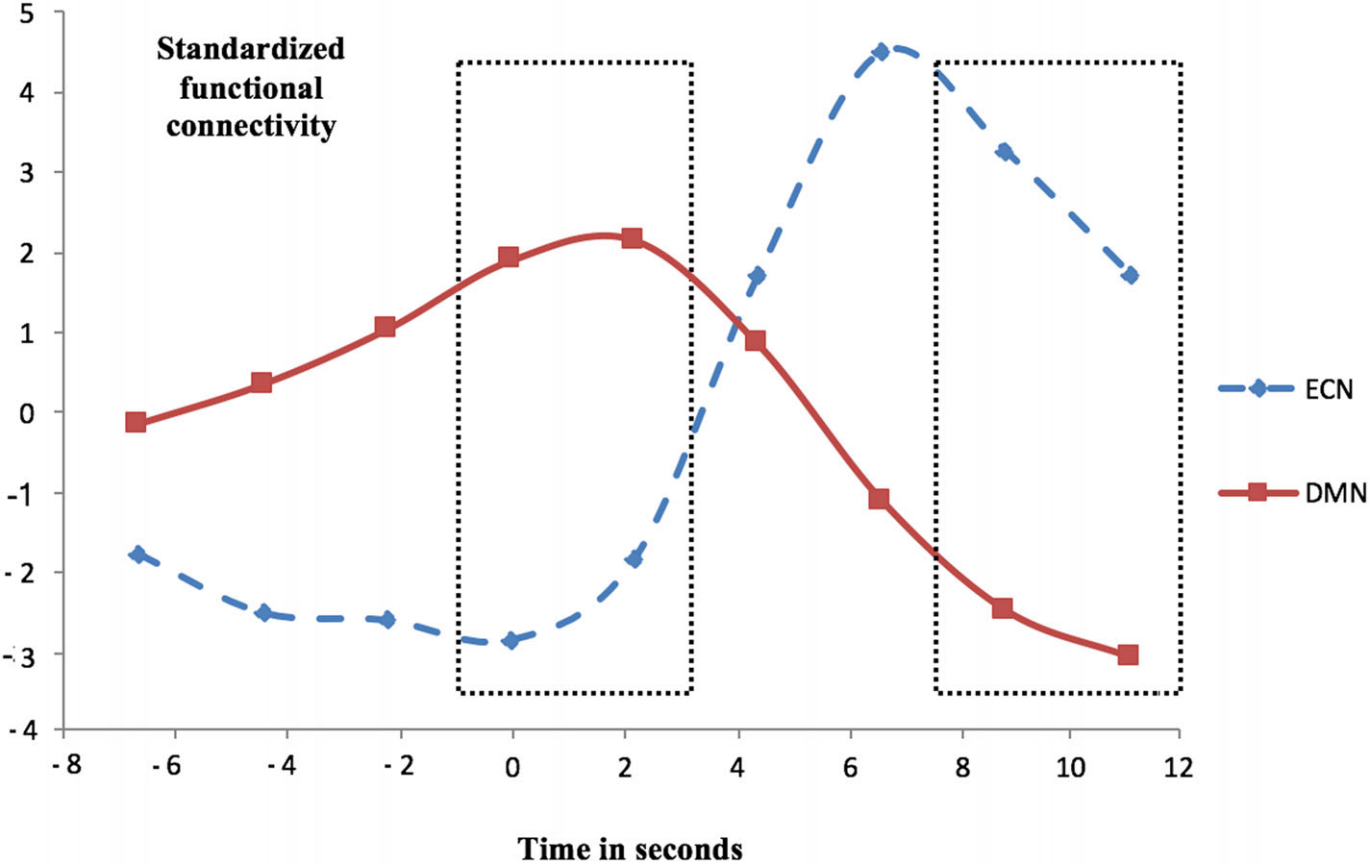
Slopes of normalized functional connectivity over time for the executive control network (ECN) and the default mode network (DMN). Rectangular frames highlight the epochs of interest. After correction for the HRF delay, the button press occurs at 6 seconds. The *y*‐axis shows the demeaned beta values resulting from the first stage of the dual regression, representing functional connectivity.

One participant had DMN normalized functional connectivity values over all time points of more than 3 SD above the mean, and was therefore removed from all further analyses involving fMRI data. Averages for the DMN and ECN were calculated for the MW (pre) and focused (post) periods, and subjected to a 2 (time) × 2 (networks) repeated measures (RM) ANOVA on DMN and ECN functional connectivity during MW (pre) and focused (post) periods. No main effect was found for time *F*(1,25) = 0.89, *P* = 0.354, *η_p_* ^2^ = 0.04; however, there was a main effect for networks, *F*(1,25) = 5.78, *P* = 0.024, *η_p_* ^2^ = 0.19, with activity greater in ECN than DMN (Fig. 2). A significant interaction effect was found between time and networks; *F*(1,25) = 31.04, *P* < 0.001, *η_p_* ^2^ = 0.55. As seen in Figure 2 and confirmed by post‐hoc *t*‐tests, DMN functional connectivity was significantly higher during MW (pre) than focused (post) episodes; *t*(25) = 5.59, *P* < 0.001, *d* = 1.10, whereas ECN functional connectivity was significantly lower during MW (pre) compared with focused (post) episodes; *t*(25) = −4.66, *P* < 0.001, *d* = 0.92. This supports hypothesis III.

### Hypothesis IV: relation between MW‐related EEG and fMRI changes

The 2 (time) × 2 (networks) RM ANOVA from hypothesis III was repeated, with the MW‐related frontal TBR change (computed as MW minus focused attention frontal TBR) added as a covariate into the model to assess if MW‐related TBR is related to MW‐related connectivity. A significant interaction effect was found for time × networks × MW−related frontal TBR changes; *F*(1,23) = 7.01, *P* = 0.014, *η_p_* ^2^ = 0.23.

**Figure 3:**
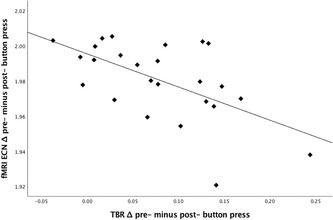
Scatterplot of the significant relation between the MW‐related changes in frontal EEG theta/beta ratio (TBR; *x*‐axis) and the corresponding changes in ECN functional connectivity (*y*‐axis); *r*(23) = −0.58, *P* = 0.002. Spearman's ranked order correlation (insensitive to outliers) was also significant; Spearman's *r*(23) = −0.54, *P* = 0.006. The plot shows log‐transformed data.

To further investigate this relation, post‐hoc Pearson correlations were calculated between the frontal MW–related TBR change scores and the corresponding difference scores (MW minus focused attention) for the functional connectivity in DMN and ECN. No association was found between the MW‐related changes in both frontal TBR and DMN functional connectivity; *r*(23) = 0.30, *P* = 0.15. However, a significant correlation was found between the MW‐related changes in both frontal TBR and ECN functional connectivity; *r*(23) = −0.58, *P* = 0.002. Figure 3 displays the scatterplot of the latter correlation, and visual inspection suggested that this relationship may have been driven by one or two influential data points. We, therefore, repeated each analysis using Spearman's rank‐order correlation, which although less powerful is more robust against such influences.^52^ The outcomes supported the results from the Pearson correlations; the MW‐related changes in both frontal TBR and DMN functional connectivity were again nonsignificant (*r*(23) = 0.19, *P* = 0.36), while a significant correlation was found for the MW‐related changes in both frontal TBR and ECN functional connectivity; *r*(23) = −0.54, *P* = 0.006. These outcomes support hypothesis IV in relation to the ECN, but not for the DMN.

### EEG and fMRI pre‐ and post‐differences related to the number of button presses

As differences were found pre‐ versus post‐button press, we explored whether these differences were related to the number of button presses. Correlational analysis showed a significant correlation between the number of button presses during EEG and during fMRI; *r*(23) = 0.49, *P* = 0.01. No significant correlations were found between the number of button presses during EEG measurement and frontal TBR difference score; *r*(24) = 0.12, *P* = 0.55 or between the number of button presses during fMRI measurement and the difference scores in DMN functional connectivity; *r*(24) = −0.24, *P* = 0.24, or ECN functional connectivity; *r*(24) = 0.24, *P* = 0.23. Thus, MW‐related EEG and fMRI change were independent of the number of button presses.

### Reported thoughts during MW episodes

Analyses of the debriefings showed that the majority of participants reported engaging in a task unrelated and stimulus‐independent thoughts during their pre‐button press episodes. During the first (EEG) session, 22% reported thoughts about the experimental procedure (e.g., worrying about losing count or moving too much) and 78% reported engaging in MW about day‐to‐day issues and episodic memories. During the second (fMRI) session, this was 19% and 81%, respectively. One participant reported distraction by the auditory oddball stimuli.

## Discussion

The aim of this study was to investigate the relations between resting state and MW‐related TBR, self‐reported AC, and their neurobiological underpinning in terms of ECN and DMN connectivity. Looking at MW operationalized as reduced sustained attention on task performance, we found that resting‐state TBR was related to increased TBR during MW. Furthermore, DMN connectivity was higher and ECN connectivity was lower during MW. For ECN, this process‐related difference was related to the process‐related difference in TBR.

TBR during rest was first associated with ADHD,^1^, ^3^ and later linked to various psychological functions and cognitive/emotional processes that rely on executive cognitive control, including trait and state AC, reversal learning, WM training, and control over automatic attentional threat biases.^10^, ^11^, ^12^, ^13^, ^14^, ^15^, ^16^, ^17^, ^18^ The current results support the hypothesis that these relations reflect TBR dynamics occurring during the resting‐state measurement that are caused by fluctuations in the balance between cognitive control and associative thought. Debriefing revealed that participants were mostly involved in thoughts about day‐to‐day issues and episodic memories or performance‐interfering thoughts about experimental procedures when they lost count, confirming that the pre‐button press periods represent periods of MW and thus loss of sustained attention toward the breath‐counting task.

TBR reflects theta and beta activity. The known functions of these two bands are in line with the current findings. Theta power has, for example, been related to decreased vigilance.^53^, ^54^ Beta is involved in behavioral inhibition,^55^, ^56^ inhibitory motoric processes,^57^, ^58^ and other controlled cognitive processes, such as WM, visual attention,^59^, ^60^, ^61^, ^62^ and attentional vigilance.^63^ These lines of evidence support the conjecture that TBR reflects an interplay between top‐down EC (beta) and activity in limbic, partially subcortical areas (theta: see Refs. 18, 64, and 65). This fits with functional correlates of TBR and its role in MW, conceived here as a state of reduced executive cognitive control and uncontrolled self‐generated thought.^24^, ^25^, ^34^, ^40^, ^64^, ^65^ Our additional finding of increased alpha during controlled attention also indicates increased involvement of top‐down processes during these periods, as alpha activity has been involved in inhibitory processes and AC over sensory information.^66^, ^67^ As beta activity is similarly involved in top‐down executive processes, both bands might have some overlap in functionality, explaining their similar increase during controlled thought periods in the current study.

The current data additionally show that the difference score of TBR during controlled versus uncontrolled thought was positively correlated to a baseline measure of resting‐state TBR (as a statistical trend, but note that one‐sided testing of this directional hypothesis would seem appropriate and would confirm the hypothesis). Whereas resting‐state TBR previously remained a “black box,” we suggest that people with less cognitive control experience more frequent and/or more profound states of uncontrolled thought during the typical EEG measurements of several minutes at rest, as individual differences between MW and cognitive control are correlated.^24^, ^25^, ^40^, ^64^, ^65^ Because the relation between resting‐state TBR and the MW‐related TBR increase was only marginally significant, this hypothesis needs to be revisited in a study with a larger sample. Unexpectedly, the often‐reported negative correlation between TBR and self‐reported (trait) AC was currently not observed and our mediation hypothesis could not be tested. The observed positive correlation between MW‐related and resting‐state TBR does, however, support the likelihood of this hypothesis, and future studies should revisit this particular test of our hypothesis. Several factors might explain the current null‐finding for the relation between TBR and AC. Participants were preselected on fMRI inclusion criteria and more than half of the samples were male, whereas previous participant samples were predominantly female. Furthermore, a positive relation between trait anxiety and baseline TBR was found, which contradicts the occasionally found negative relation between these two variables (see Refs. 10, 14, and 15) and suggests that the current sample differed in some relevant aspect from previous samples. Alternatively, the eyes‐closed only TBR assessment in this study (resting‐state TBR is typically based on eyes‐open and ‐closed measurements) might explain this null‐finding for the TBR–AC relation.

Our fMRI data during the same task, collected on another day, showed that functional connectivity in the ECN was lower during MW compared with controlled thought periods and that connectivity in the DMN was higher during MW compared with controlled thought periods. The DMN includes the PCC, mPFC, PHG, and AG. Functional activity and connectivity within this network was found to be high during task‐unrelated thoughts,^32^ and also to directly relate to MW.^38^, ^39^ Also, a recent study of Delaveau *et al*.^41^ found that depressed out‐patients had a decreased negative functional connectivity (anticorrelation) between the DMN and the salience‐network when ruminating, as compared with focused control. They also found an increased anticorrelation between the DMN and the so‐called task‐positive network during focused control. The latter network is functionally related to the ECN and involves WM processes and attention directed to the external world. The ECN that was observed in the current study showed stronger functional connectivity after than before the button press. The ECN that was selected for this study was as defined by Smith *et al*.,^51^ and covers several frontal areas, including the dorsolateral PFC (dlPFC), ACC, and paracingulate cortex. This ECN is based on a broad scope of prior (fMRI) research defining EC. fMRI studies showed that areas like the lateral PFC, dlPFC, ACC, inferior frontal junction, and parietal regions are all involved in EC functions as described by Miyake *et al*.:^68^ attentional inhibition and shifting, and the updating of WM representations.

Crucially, changes in EEG dynamics in this study were related to fluctuations in the ECN, which, for the first time, directly supports the notion that TBR dynamics are related to functional connectivity in brain networks involved in executive cognitive control (the relation between TBR change and DMN change of a near medium effect size was in the predicted direction, but nonsignificant). The fMRI results from our study thus demonstrate that the transition between MW episodes and episodes of controlled thoughts (and metacognitive awareness), as measured with the breath‐counting task, is associated with increased connectivity between brain areas that have been convincingly shown to be crucial for AC and executive cognitive processing. The observed relation between MW‐related changes in TBR and ECN functional connectivity strengthens previous conceptualizations of TBR as reflecting voluntary top‐down processes of EC (including AC), mediated by dlPFC, over bottom‐up processes from limbic areas.^13^, ^15^, ^69^, ^70^, ^71^ For instance, recent studies from our laboratory reported that TBR moderated automatic attentional threat‐biases as measured by a dot‐probe task,^14^, ^15^ and by an emotional threat interference task^16^ in the manner predicted by theories explaining the role of catecholamines in PFC‐mediated executive functioning,^72^, ^73^ and theoretical models describing the role of cognitive control over such automatic attentional biases to threat (see Refs. 74 and 75). It has been suggested that exposure to such acute threat prompts a reallocation of resources to the salience network at the cost of the ECN.^76^ Also, worry (noticeably increased in affective disorders of anxiety and depression; see Refs. 77 and 78 for reviews) represents biased internal activation of threatening cognitions in WM, and shares mechanisms with biased attention.^79^ Worry can be seen as self‐generated off‐task thought, and is sometimes referred to as a “negative form” of MW.^23^ Our current findings support the suggestion that TBR's role in the regulation of automatic attentional threat bias reflects such interplay between bottom‐up, mainly subcortical, and top‐down prefrontal cortical networks,^76^ as first suggested by van Honk and Schutter^18^ and Knyazev,^69^ and supported by various studies of our own and other laboratories.^10^, ^11^, ^12^, ^13^, ^14^, ^15^, ^16^, ^17^, ^18^, ^19^, ^20^, ^22^, ^55^, ^80^, ^81^, ^82^ Our current findings further underline the importance of TBR in executive functions and its possible applicability when investigating these. TBR may be used as a marker of MW‐related changes in brain activity and can likely be very useful for the study of MW^30^ and inattention.^83^, ^84^

To approximate the procedure of Braboszcz and Delorme^33^ as closely as possible, (oddball) auditory stimuli were presented during the breath‐counting task. Such stimulus presentation has been reported to increase theta and decrease beta activity.^33^, ^85^ However, auditory stimuli were presented in a random timing and were predicted a priori to occur approximately equally often during the epochs before and after the button presses. They are thus unlikely to have systematically biased comparisons between epochs, although they might have caused some unsystematic noise. Now that the basic method of relating TBR to MW in this task has been firmly replicated twice, future studies could omit the auditory tones from the procedure.

Interestingly, the ERSP‐derived spectral plot and the functional connectivity plot (Figs. 1 and 2) revealed that after a “drop” that started just before the button press, already within the post‐button press window of ∼6 s, TBR seems to be going up again, and also connectivity of ECN seems to quickly return toward pre‐button press values. For EEG, this was previously observed,^34^ and explorative post‐hoc tests (not reported) confirmed this temporal pattern for TBR/ECN connectivity. This could possibly indicate that individuals start to lapse back into a new MW episode again within our defined window of 1.7 to 6.1 s after the button press, but that seems unlikely, so shortly after their becoming aware of MW. A potentially more interesting speculation is that the focused periods (controlled thought) might represent a short hypervigilant meta‐awareness (realizing that one lost count and was MW, and subsequently increasing the use of executive resources for goal‐directed monitoring of breath counting), contributing to the frontal TBR change pre‐ versus post‐button press (see also, van Son *et al*.^34^). This would be in line with the literature on EEG changes in theta and increased hypervigilance after error realization.^86^, ^87^ Future studies could take this speculation into account by examining a shorter post‐button press period.

A potential limitation of this study is that the EEG and fMRI measurements took place several days apart (*M* = 2.8 days). Simultaneous testing of EEG and fMRI would be even more powerful. However, the fact that we did find the predicted correlation between changes in TBR and fMRI measures validates the robustness of our method, and of TBR and its functional neural correlates. Another issue is that the breath‐counting MW method as used in this study and in van Son *et al*.^34^ (see also, Ref. 33) has the potential limitation of relying on introspection. Since the MW episodes that are examined are self‐reported and in close temporal proximity of this self‐reported awareness, their underlying brain activity might not represent all MW‐related brain activity. Future studies might correlate EEG and/or connectivity dynamics of this method with methods of probing MW that do not solely rely on self‐report. On a related note, it might be argued that participants who were better capable of detecting their own MW episodes pressed the button more often, resulting in data being driven by these participants. This could then potentially imply that our findings are not similarly representative for people with good versus poor meta‐attentional introspective awareness. However, this alternative explanation seems ruled out by the absence of significant correlations between the numbers of button presses and the observed effects of MW on EEG and fMRI measures. Finally, since data with too few artifact‐free epochs were excluded from analysis, reported results are for quite a small participant subsample, biased toward a high MW episode count during the task, likely reflecting higher than average trait MW. Generalization of the current results is thus likely limited to people who often engage in MW and/or people with reduced sustained attention capabilities.

In sum, the present study importantly contributes to research into TBR as an electrophysiological marker of EC. Our findings provide clear indications of the neuropsychological functional nature of TBR as well as its neural underpinnings, something that was much needed after several decades of TBR research. This increases our understanding of TBR's relation to psychiatric symptomatology and more firmly establishes frontal TBR as a useful and easy, low‐cost tool in the study of EC in normal and abnormal psychology.

## Author contributions

All authors were involved in the study design and approved the final manuscript.

## Competing interests

The authors declare no competing interests.
